# Detection of Severe Respiratory Disease Epidemic Outbreaks by CUSUM-Based Overcrowd-Severe-Respiratory-Disease-Index Model

**DOI:** 10.1155/2013/213206

**Published:** 2013-08-28

**Authors:** Carlos Polanco, Jorge Alberto Castañón-González, Alejandro E. Macías, José Lino Samaniego, Thomas Buhse, Sebastián Villanueva-Martínez

**Affiliations:** ^1^Facultad de Ciencias de la Salud, Universidad Anáhuac, Avenida Universidad Anáhuac No. 46, Col. Lomas Anáhuac, 52786 Huixquilucan, MEX, Mexico; ^2^Subdirección de Epidemiología Hospitalaria y Control de Calidad de la Atención Médica, Instituto Nacional de Ciencias Médicas y Nutrición Salvador Zubirán, Vasco de Quiroga 15, Piso 4, Col. Sección XVI, DF, México 14000, Mexico; ^3^Departamento de Matemáticas, Facultad de Ciencias, Universidad Nacional Autónoma de México. Cd. Universitaria, DF, México 04510, Mexico; ^4^Centro de Investigaciones Químicas, Universidad Autónoma del Estado de Morelos, Avenida Universidad 1001, 62209 Cuernavaca, MOR, Mexico

## Abstract

A severe respiratory disease epidemic outbreak correlates with a high demand of specific supplies and specialized personnel to hold it back in a wide region or set of regions; these supplies would be beds, storage areas, hemodynamic monitors, and mechanical ventilators, as well as physicians, respiratory technicians, and specialized nurses. We describe an online cumulative sum based model named Overcrowd-Severe-Respiratory-Disease-Index based on the Modified Overcrowd Index that simultaneously monitors and informs the demand of those supplies and personnel in a healthcare network generating early warnings of severe respiratory disease epidemic outbreaks through the interpretation of such variables. A *post hoc* historical archive is generated, helping physicians in charge to improve the transit and future allocation of supplies in the entire hospital network during the outbreak. The model was thoroughly verified in a virtual scenario, generating multiple epidemic outbreaks in a 6-year span for a 13-hospital network. When it was superimposed over the H1N1 influenza outbreak census (2008–2010) taken by the National Institute of Medical Sciences and Nutrition Salvador Zubiran in Mexico City, it showed that it is an effective algorithm to notify early warnings of severe respiratory disease epidemic outbreaks with a minimal rate of false alerts.

## 1. Introduction

A future severe febrile respiratory illness outbreak will affect a large number of people; many of them will suffer from acute respiratory failure, demanding critical care by specialized personnel with equipment to meet the demand of what is call now “mass casualty mechanical ventilation.” This scenario can appear simultaneously in distant locations without any apparent relationship between them [1, 2], replicating itself very fast in the region. In this scenario, the potential benefit from using automated monitoring methods in a healthcare network is highly promising [3, 4] specially if in addition to monitor the variables involved it supports decisions with “fresh” online data during the outbreak to optimize medical supplies.

In an epidemic outbreak, the health sector needs to know accurately and in an extremely short period of time the outbreak location, the region or regions where it is spreading, its propagation speed, and the human workforce and supplies available and their location (Table 1) [5]. Although hospital facilities have updated inventory lists, protocols, and agreements to share specialized personnel and supplies in case of an epidemic outbreak [6], most of them do not have figures from other hospital inventories because this information is centralized in health sector government offices [7], making it difficult to build possible transit scenarios of human and material supplies in the network; precious time is thus lost, and medical care is delayed during these emergencies [8]. From these facts, we gather that if every hospital facility has updated information about the entire network, it will empower them to take earlier decisions about the optimal distribution of resources at the time that the epidemic outbreak is detected. We believe that the demand of these supplies is a parameter that correlates with the respiratory epidemic outbreak itself. Accordingly, the demand of these supplies in disasters is not unique of a respiratory epidemic outbreak, however, the circumstances in time and space definitely are. 

To validate our idea, we developed an *online* model named Overcrowd-Severe-Respiratory-Disease-Index (OSRDI), which was inspired by a Modified Overcrowd Index system used to measure the attention in the Emergency Department [5]. The index does not need large computational background, shares the data of each hospital facility for the entire network, and identifies in which cumulative sum (CUSUM) algorithm [9] there is an unusual demand of the aforementioned supplies. With the information, the model warns over possible respiratory epidemic outbreaks when the number of warnings by area unit exceeds the parameters described later. This system is not predictive; it only gives a fast count that could improve predictive models [10, 11] as there is a *post hoc *analysis of the evolution of those variables which provides elements to understand the propagation of the epidemic. We think that this model helps to solve the problem of personnel and material shortages in the network created by an epidemic outbreak, as it enables the network to reallocate in real time physicians and supplies, particularly—but not limited—to the Emergency Department and the Intensive Care Unit, supporting the alert that the system gives and optimizing time and resources in the affected areas. It also provides recommendations for the final distribution and logistics of these resources, which can be considered an additional asset over other models already known. 

## 2. Materials and Methods

We developed an algorithm to monitor daily reported needs of materials and specialized personnel in a network of healthcare facilities. The model was evaluated using simulated multiple outbreaks superimposed on historical baseline data and the 2009 H1N1 influenza outbreak in México [12–16], with figures taken from the National Institute of Medical Sciences and Nutrition Salvador Zubiran (INNSZ) census. Later there is a description of the model, scenario evaluation, algorithms, and performance indicators. As said before, the model was inspired by the Modified Overcrowd Index, recently developed by our group [5].

### 2.1. Overcrowd-Severe-Respiratory-Disease-Index (OSRDI)

The OSRDI model is a CUSUM [9] algorithm. The CUSUM-based calculation means the calculation of a cumulative sum. Samples from a process *x*_*n*_ have been assigned weights *w*_*n*_ and summed as follows:

(1) $$\begin{array}{c} S_{0}=\left(\;\right),\\ S_{n+1}=\max\left(0,S_{n}+x_{n}-w_{n}\right). \end{array}$$

With this formula, the OSRDI model weights the variables described in Table 1, detecting any unusual consumption of resources in the entire hospital network, generating warning alerts in the facility where it is located and in nearby region facilities as the algorithm builds up specific areas by postal code. This space distribution varies depending on the fluctuation of the network nodes. The method considers four quotients in two different levels of information to give a warning from the variables mentioned previously. The first three quotients are given before the outbreak.

1. 100 × (available beds)/(doctors + respiratory technicians + nurses). This saturation rate gives the relation between available beds and doctors, respiratory technicians and nurses. The quotient is a referential of the attention given to patients hospitalized by the specialized healthcare personnel. If there are no doctors, respiratory technicians, or nurses at the time of taking the inventory, the quotient will be calculated as 999; being so, the range of the quotient fluctuates between 0 and 999.
2. 100 × (available areas)/(hemodynamic monitors + mechanical ventilators). This saturation rate gives the relation between available areas in the hospital facility and the equipment. This quotient is a referential of possible relative growth in the facility, considering the maximum available areas where an equipped bed can be placed with the available equipment. In case of denominator zero because of no hemodynamic monitors or mechanic ventilators available at the time of taking the inventory, the quotient will be calculated as 999.
3. 100 × (available beds)/(hemodynamic monitors + mechanical ventilators). This saturation rate gives the relation between usable beds and the equipment in the hospital. This quotient is a referential of possible relative growth in the healthcare facility considering the total beds usable and the equipment. If there are no hemodynamic monitors or mechanical ventilators at the time of taking the inventory, the quotient will be calculated as 999.

Additionally, there is one quotient given at the outbreak itself taken from the number of patients requiring a specialty bed (see Table 1) and the number of available beds in the healthcare facility.

(4) 300 × (patients)/(available beds). This saturation rate measures the attention quality in terms of the number of patients requiring a specialty bed and the equipment for the acute respiratory failure, related to the number of available beds. In case the denominator is zero, meaning there are no available beds in the facility at the time of taking the inventory, the quotient will be calculated as 999.

The model also displays two different subindexes (A and B).

- The first subindex shows the resources distribution that a respiratory disease outbreak rapidly consumes, taking the maximum quotient from 1, 2, and 3.
- The second subindex is related to the potential respiratory disease outbreak itself. Quotient (4) is pondered 30% if any quotient (1, 2, or 3) is greater than 90.

The model uses these two quotients taking the biggest. For instance, if the quotient (1) has 100 as value, quotient (2) has 165, quotient (3) has 201, and quotient (4) has 350, then the OSRDI-A would be 201 (3: extremely saturated) and the OSRDI-B would be 350 × 1.30 = 455 (see Table 2).

The model has four levels of alert used also by the Modified Overcrowd Index [5] (Table 2); levels A and B are associated with a warning message. The daily use of the system will place in context the level of alert in the hospital facility; the method will take the highest value from subindexes (A and B) at the time of the alarm, in other words, OSRDI = max (OSRDI-A, OSRDI-B). 

### 2.2. Geographical Regions

The model also takes into account the geographical area of each affiliated hospital the same way as it does in the Modified Overcrowd Index [5]. The geographical area is recognized as a region by the OSRDI applying this formula |PC_*m*_ − PC_*i*_| < 4. In this expression the symbol | | stands for the absolute value of the expression, where PC_*m*_ is the arithmetic mean of nearby hospital area codes and PC_i_ is the area code of one specific hospital in the network. The difference between those variables is 4 (|PC_*m*_ − PC_*i*_| < 4); it induces a circle with a three kilometers radius, and therefore this algorithm is able to build regions within this area.

The saturation warning will be displayed if more than 75% of the hospitals in that area reach a value higher than 100. For example, let us consider a network of 13 hospitals (Table 3) with three regions {1, 2, 3, 4}, {6, 7, 8, 9}, and {10, 11, 12} according to their area codes, hospitals 5 and 13 are distant from each of the regions, and consequently they are not part of them. 

The OSRDI model defines a region if the absolute difference between two area codes is less than 4 kilometers. If the arithmetic mean of hospital group {1, 2, 3, 4} is PC_*m*_ = 10501; and the area code of hospital 1 is PC_1_ = 10500 then |PC_*m*_ − PC_*i*_| = |10501 − 10500| = 1 < 4.

The same applies for the second hospital where PC_*m*_ = 10501 and PC_*i*_ = 10501, then |PC_*m*_ − PC_*i*_| = |10501 − 10501| = 0 < 4, and so forth until hospital 5 where |PC_*m*_ − PC_*i*_| = |10501 − 10510| = |−9| = 9 > 4. OSRDI does not consider this hospital part of the region; therefore it rejects it.

In region {1, 2, 3, 4} the level to set the warning has been reached because the highest OSRDI value is 250 corresponding to “3: Extremely Saturated,” in region {6, 7, 8, 9} the warning is “2: Highly Saturated” as the OSRDI value is 199, while region {10, 11, 12} remains below the warning level as the OSRDI value is 99. 

In the case of hospitals 5 and 13, the OSRDI reaches 300 and 400, respectively, which are displayed in the surveillance system, but they do not cause any alert because the model is adjusted to region or network but not to isolated units. The OSRDI determines the region automatically and submits the warning if required. The model constantly verifies the regions built up adding or withdrawing any hospital facility and makes the necessary adjustments. 

It is advisable to initially use the model in hospitals at the same geographical region. Although being designed for hospital networks scattered in distant regions or states, the initial setup would determine the level of alert in that particular region. 

### 2.3. Retrospective Analysis

Apart from showing quotients A and B in real time, the model keeps a historical backup of 16 quotients to enable a “time series” construction (Table 4). This backup allows an analysis of a hospital unit as well as all hospitals in the network. Those 16 quotients were next divided in four groups for simplicity.

- Quotients (5-6) show a maximum demand on equipment with regard to specialized personnel and available beds in the facility.
- Quotients (7–9) show a maximum demand of specialized personnel with regard to available beds, available areas, and equipment in the facility.
- Quotient (10) shows a maximum demand of available beds with regards to available areas in the facility.
- Quotients (11–13) show the demand of available areas with regard to patients, available beds, and specialized personnel in the hospital.
- Quotient (14–16) show the demand of patients with regard to available areas, specialized personnel, and equipment in the facility.

### 2.4. The OSRDI Screens

The model shows the following variables when updated: patients, available beds, available areas, equipment, specialized personnel, quotients A, B, and time (Table 5). The field “time” shows the last time that the data was updated. These variables appear for each hospital connected to the model, so the user can access the data of the entire network at any time and become aware of a possible saturation of other hospital services. The system also provides an audible signal, so any passive observer can also be aware of the warning.

### 2.5. Scenario Evaluation

To evaluate the model we performed two tests: a hospital census and an automated recreation of multiple epidemic outbreak scenarios generating at random all variables here described.

1. We used the 2009 census derived from the observations made to the H1N1 epidemic outbreak in 2008–2010 by INNSZ; the available beds for this purpose represented 20% of total beds, and the figures refer to patients admitted into the hospital with unknown acute respiratory syndrome.
2. The exhaustive test consists of the recreation of multiple warning alerts in a group of 13 hospitals, called built-in self-random test (BISR), as the Modified Overcrowd Index does [5].
3. For this purpose, 52,560 hospital scenarios were randomly generated by the use of a random number generator [17, 18], and the corresponding data was converted into the various variables used by the model. These random scenarios simulated hourly data readings for a time span of 6 years. This procedure was repeated for 13 different hospitals distributed in two regions. The BISR test generated variables for each hospital that were inspected by the model.
4. Reference data (“seed”) for the BISR test was taken by the INNSZ in January 2008 and was used as the base for the simulated data reading, increasing or decreasing the corresponding variables at random. An example is given in Table 6 where random percentages generated by the BISR test were applied to the variables of Table 1, then a random template was constructed that fed the model, the same way the BISR test generated all virtual scenarios.
5. For the OSRDI verification we calculated a “double blind” test on 52,580 random transactions and counted the “false negative” and “false positive” answers produced by the system.

### 2.6. Performance Indicators

The model performance was focused on multiple simulated scenarios of respiratory epidemic outbreaks, measuring the initial and final stages of each outbreak produced randomly and all reconfigurations that the model produced from the 13 hospitals. Each individual and regional warning was registered as well as the model average execution time. The triage results were used to assess the model performance and the relation response false alarm. 

The model average performance is based on two main indicators: its sensitivity, which describes the algorithm capability to detect simulated outbreaks; and the time required to give the warning. In all cases the evaluation of the warning signal was done first at the hospital and then at the region. A false warning was considered as a period with no epidemic outbreak warning. 

### 2.7. Cut-Off Points

*Quotients.* The OSRDI system is sensitive to two measures: (i) the installed capacity to deal with an outbreak of the disease, and (ii) the demand and offer of the referenced variables with respect to patients. The first three subindexes measure the variables: beds, equipment, and health care staff, while the fourth subindexes weighted heavily the availability of beds and patients who require these beds, as an indication of the beginning of an outbreak. The design of this method considers that a severe respiratory failure may occur with the need of immediate hospitalization, but a second indicator may come from an unusual demand for supply, on a specific region.

This method assumes that a considerable number of hospitals are added to the system to form a hospital network, and a consequence of this is the common knowledge of all users on the real demand of supplies involved. It is in this sense that the imbalance between the different variables (Table 1) is indicative, not only of a possible outbreak but also of the shortcomings of equipment for hospitals.

*Scenario Evaluation.* The test was performed in the National Institute of Medical Sciences and Nutrition Salvador Zubiran, because the data available to us and the system depend on the constant update of inventories and personnel, and not the type of hospital. The testing of the automated system envisioned the adequacy of a virtual stage that was also used in another system [5], and it was very effective simulating years of processing. We used a method implemented and tested by us for the random generation of events [18], with computers dedicated solely for the processing of these tests (see Section 6). We did not limit the random values to much more probable ranges (see Table 6, column respiratory technicians), since the idea was to validate even events that would be very unlikely.

*Geographical Regions.* To assign a region, after the design of the OSRDI system was the most elaborate and difficult part, considering that when one identifies a region on a map, one identifies it visually. However, to set up a rule and then automate it is radically different. In addition, the OSRDI analytically identifies several regions at the same time and updates them according to the addition/removal of hospitals. We believe that if 75% of the region presents a saturation index greater than 100, it is a symptom of a disease outbreak, and that although increasing the number of affiliated hospitals will benefit from the knowledge of the situation that the supplies have at that moment, we do not consider that this will add efficiency to the predictions, since the phenomenon that seeks to predict is multifactorial. The cutoff value of 75% was selected because we have seen that most of the Emergency Departments in a metropolitan area work with high occupancy indicators under normal conditions.

## 3. Results

**Figure 1:**
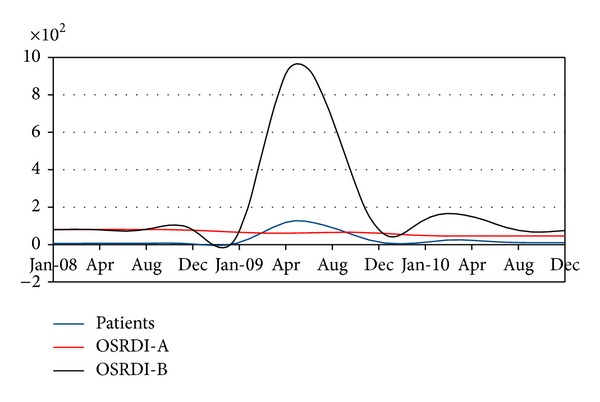
OSRDI-A and OSRDI-B sensibility to patient distribution in census taken by INNSZ in Mexico City from 2008 to 2010 (Table 7).

Table 7 shows the variable trend registered by the INNSZ from 2008 to 2010 (rows 1–8), as well as the OSRDI estimated saturation index (row 11). The April 2009 outbreak was detected by the model only from the unusual growth in the number of patients as the other variables did not change during this event. The model sensitivity is graphically illustrated (Figure 1) by comparing patients' linear trend in the census versus the A and B indexes. The graphic shows the model sensitivity at the strong fluctuation of the patient variable. There is practically no change in the OSRDI-A index while the OSRDI-B has a significant change, showing the need to include all variables in the four quotients to generate the outbreak warnings. 

Table 8 shows the warning coincidences generated at random by the six-year simulation in 13 hospitals compared to the warnings generated by the model, divided into two initial groups by the different variables (available beds, usable areas, and specialized medical personnel and equipment). All variable warnings match, except the number of usable areas; in this case, the model did not result in a warning. 

### 3.1. Robustness

The model has been designed to be accessed from any location, with an unlimited number of hospitals and users. Its access communication port is 22 with firewall protection against other computers. Its security system has an automated scaled backup with reports on a daily, weekly, and fortnightly basis. The backups are online and offline for all hospitals in the network, and it also has a connectivity failure detector; in case of 35% connectivity loss it will send an alarm to the administrator. The system has fragmented access, encrypted and distributed in several servers; some of them are also randomly placed offline.

The OSRDI model has been designed to use minimal computer resources and can be configured to start when the equipment is turned on, the epidemic outbreak warning alarm can also be audible if requested. 

### 3.2. Availability

Tests carried out for implementation by INNSZ show that data variable input (Table 1) should be done by the Intensive Care Unit and the Emergency Department and subsequently by the hospital admission area. The use of the model only for monitoring purposes should be in the hospital epidemiology department. The historical information (Table 4) should be easily available but only within the hospital network. All warning alerts must be confirmed by the highest authority in the health sector. To avoid data input duplication, the hospital can use patient admission records as parameters (Table 1) [5]. At this stage a hospital can use the model to find out the saturation index in its patient first-contact services.

## 4. Discussion

During the H1N1 Influenza epidemic outbreak, difficulties were faced to solve the cases of respiratory distress that hundreds of patients suffered. In some cases, proper areas were available in the facility, but beds or hemodynamic monitors were lacking; in others, mechanical ventilators were at hand but not the suitable staff to use them. Considering that in case of a disaster, resources are always limited to the circumstances and that respiratory distress is the first failure that people in critical condition face, it is expected that in any severe respiratory disease outbreak the demand of this kind of equipment, material, and staff will be dramatically increased. Based on these facts, the computational OSRDI model was designed to assist the healthcare system at three stages.

- Previous to the outbreak, inventorying the equipment in the hospital facility so the model can recommend the transference of basic equipment, material, and personnel to optimize their use and to avoid shortage of them.
- During the epidemic outbreak taking record of any unusual shortage of supplies and reporting it to the facility.
- After the event reporting the saturation level of the hospital network. The model does not identify possible internal logistic problems in hospitals whether public, private, or specialized; it does not discriminate between different hospital locations. It can also be used to give support to land or air transportation of patients providing in advance the availability of beds and personnel in the area before the patient arrives to the hospital.

The OSRDI model is a fast count algorithm whose mathematical simplicity makes it easy to implement; however, it is not a predictive algorithm. Some models that predict the outbreak based on symptoms are the PDE algorithms [19] that use partial differential nonlinear equations or the HMM [20] algorithms based on Hidden Markov models. PDE-based algorithms are very restrictive as the resolution of the differential system grows in its complexity with an *X* ^*X*^, factor where “*X*” is the number of parameters involved. The HMM based algorithms require precise training profiles, which is a drawback as the nonspecific nature of the symptomatology in respiratory outbreaks makes it difficult to identify. 

The algorithms aimed at the automated monitoring of epidemic outbreaks of influenza, such as EARS algorithms that use diagnostic and prediagnostics information. However, it is very difficult to avoid false alarms due to the fact that the outbreaks accuse multiple variables, so that small differences induce changes in the fundamental values of prediction. Although our model cannot account for the details and internal complexity of hospital saturation, its outline can immediately identify such scenario in time and space. 

The suggestions made by the model, for instance, the transfer of equipment, have to be finally authorized by qualified staff; however, these suggestions can become vital in the case of an emergency for its logistic implications. We consider that the generalized use of the OSRDI model by the health sector could be a valuable contribution to solve complex situations when facing an epidemic outbreak. 

## 5. Computational Platform

The model was written in Fortran 77 under a Unix-type operating system (GNU) and was performed with an Intel i686, 0.5 GB of memory, 100 GB total storage memory and a Linux Fedora 14 operative system. This platform allowed the measurement and verification of each routine of the model. This task is always important in modeling, but in this particular case it was more relevant as it was necessary to verify all routines that generated random numbers implemented and whose methodology had been previously used by us [18]. 

The reason to verify the generation of random numbers does not concern the verification quality of the set of randomly generated numbers but the quantity (or periodicity) of those random numbers produced. A procedure generating random numbers relies on a computer that has a limited representation in whole numbers and decimals. Hence even if the generator can be mathematically tested to have an infinite periodicity, it will never be so in a finite computer. Therefore, if random number generators are not checked in a process where the number of interactions exceeds the generator large sequences, the generator repeats the same sequence or part of the same sequence, consequently the randomness of the process is lost.

## 6. Conclusion

The computational system named OSRDI is an effective and practical algorithm that detects potential severe febrile respiratory illness epidemic outbreaks in hospitals by measuring few variables. This model features a high efficiency to exclude false alarms.

## Figures and Tables

**Table 1:** OSRDI model variables.

No.	Concept	Description
1	Patients	Number of patients in the healthcare facility according to the following description. Description: a patient already admitted into hospital requiring a bed, the equipment, and the attention of specialized personnel.

2	Available beds	Number of beds in the healthcare facility according to the following description. Description: a bed in good condition and maintenance that provides comfort, supplied with necessary clothing. The headboard must be in contact with the wall, away from windows or doors that can obstruct hospital personnel free transit; beds must be articulated, their height must be adjustable and easy to handle and move, and they have to be provided with wheels; other fixtures and fittings would be a hypoallergenic waterproof mattress breathable to water steam, side rails, pillow with pillowcase, handle to adjust height and articulate the bed. Each bed must have a free space that has an air, oxygen, and suction outputs, as well as an easily available electricity supply outlet. Hospital bed dimensions: 0.80–0.90 m. (width) × 1.80–1.90 (length) × 0.70 m. (height without mattress). Minimal distance between beds: 1.20 m.

3	Available areas	Number of physical areas available in the healthcare facility, according to the following description. Description: an available area to place a bed with infrastructure and the necessary equipment to give permanent assistance and clinic monitoring of vital signs to patients in critical condition. This area must have easy access to hospital, emergency department, intensive care unit, intermediate care facilities, and have air, oxygen, and suction outputs and an electricity supply outlet. The electrical installation must have 120 V to 240 V and easily identifiable ground connections.

4	Hemodynamic monitors	Number of usable hemodynamic monitors in the healthcare facility according to the following description. Description: any monitor that measures patients' hemodynamic parameters. Compulsory equipment: continuous electrocardiograph monitor (ECG) in one or two leads (DII and V5), continuous heart rate monitor, pulse oximeter (SpO_2_), noninvasive blood pressure monitors at 10 min intervals. Optional equipment: Capnographs, exhaled CO_2_ monitor (EtCO_2_), ST analysis, and invasive blood pressure monitors.

5	Mechanical ventilators	Number of usable ventilators in a hospital facility according to this description. Description: any mechanical ventilator with the following modes: assist control ventilation (ACV), synchronized intermittent mandatory ventilation (SIMV), and pressure support ventilation (PSV), with variable control (pressure, volume, flow, or time) and dual control, PEEP with battery to store energy, and a pneumatic compressor for adult and pediatric patients.

6	Doctors	Number of doctors in the hospital facility according to this description. Description: doctors with postgraduate degree and/or training with patients in critical condition and/or anesthesiology, attested by a certified university program.

7	Respiratory technicians	Number of respiratory technicians in the hospital facility according to the following description. Description: respiratory technicians or personnel trained to treat patients on mechanical ventilation.

8	Nurses	Number of nurses in the hospital facility according to the following description. Description: nurses or technicians trained to treat patients in critical condition and surgical emergencies.

Variable description used for OSRDI model to determine saturation index (Section 2.1), [5].

**Table 2:** Main saturation stages.

No.	Level	Description
3	*x* > 200	Extremely saturated
2	100 < *x* < 200	Highly saturated
1	100	Saturated
0	*x* < 100	Normal

Level: acceptance range for OSRDI saturation index (Section 2.1) [5].

**Table 3:** Warning rate for an OSRDI network.

Hospitals	1	2	3	4	5	6	7	8	9	10	11	12	13
Area codes	10500	10501	10500	10503	10510	10988	10987	10985	10986	10300	10301	10303	10600
OSRDI	150	200	250	110	300	199	189	170	165	50	60	99	400
Warning rate	250	250	250	250		199	199	199	199				

Example of warning system in a 13-hospital network divided by two regions. Hospitals: 13 hospitals identified by area code. Area codes: location identifiers for the 13 hospitals. OSRDI: saturation index calculated by the model. Warning rate: saturation rate by region (Section 2.2).

**Table 4:** OSRDI backup model.

No.	Concept	Description
1	Patients	Number of patients in the healthcare facility.
2	Available beds	Number of beds in the healthcare facility.
3	Available areas	Number of physical available areas in the healthcare facility.
4	Hemodynamic monitors	Number of usable hemodynamic monitors in the facility.
5	Mechanical ventilators	Number of usable mechanical ventilators in the healthcare facility.
6	Doctors	Number of doctors in the healthcare facility.
7	Respiratory technicians	Number of respiratory technicians in the healthcare facility.
8	Nurses	Number of nurses in the facility.
9	Quotient (1)	(Available beds)/(doctors + technicians + nurses).
10	Quotient (2)	(Available beds)/(hemodynamic monitors + mechanical ventilators).
11	Quotient (3)	(Available areas)/(hemodynamic monitors + mechanical ventilators).
12	Quotient (4)	(Patients)/(available beds).
13	Quotient (5)	(Hemodynamic monitors + mechanical ventilators)/(doctors + technicians + nurses).
14	Quotient (6)	(Hemodynamic monitors + mechanical ventilators)/(available beds).
15	Quotient (7)	(Doctors + technicians + nurses)/(available beds).
16	Quotient (8)	(Doctors + technicians + nurses)/(hemodynamic monitors + mechanical ventilators).
17	Quotient (9)	(Doctors + technicians + nurses)/(available areas).
18	Quotient (10)	(Available beds)/(available areas).
19	Quotient (11)	(Available areas)/(patients).
20	Quotient (12)	(Available areas)/(available beds).
21	Quotient (13)	(Available areas)/(doctors + technicians + nurses).
22	Quotient (14)	(Patients)/(available areas).
23	Quotient (15)	(Patients)/(doctors + technicians + nurses).
24	Quotient (16)	(Patients)/(hemodynamic monitors + mechanical ventilators).
25	Date	It is the date the healthcare facility updates the data.
26	Overcrowd-Severe-Respiratory-Disease-Index model A	It is the OSRDI-A computation.
27	Overcrowd-Severe-Respiratory-Disease-Index model B	It is the OSRDI-B computation.
28	Time	It is the moment the healthcare facility updates the data.

Concept: OSRDI model backup that adds up on each update done by the user (Section 2.3).

**Table 5:** OSRDI screen.

No.	Concept	Description
1	Patients	Number of patients in the hospital facility.
2	Available beds	Number of beds in the hospital facility.
3	Available areas	Number of physical available areas in the hospital facility.
4	Hemodynamic monitors	Number of usable hemodynamic monitors in the hospital facility.
5	Mechanical ventilators	Number of usable mechanical ventilators in the hospital facility.
6	Doctors	Number of doctors in the hospital facility.
7	Respiratory technicians	Number of respiratory technicians in the hospital facility.
8	Nurses	Number of nurses in the hospital facility.
9	Quotient (1)	(Available beds)/(doctors + technicians + nurses).
10	Quotient (2)	(Available beds)/(hemodynamic monitors + mechanical ventilators).
11	Quotient (3)	(Available areas)/(hemodynamic monitors + mechanical ventilators).
12	Quotient (4)	Patients/(available beds).
13	Time	It is the moment the data is updated.

Concept: variables shown in the screen by OSRDI model after each update (Section 2.4).

**Table 6:** Built-in self-random-test parameters.

Date	Patients	Available beds	Available areas	Hemodynamic monitors	Mechanical ventilators	Doctors	Respiratory technicians	Nurses
Jan-08	6	191	68	33	52	25	13	67
Built-in self-random test	−5%	10%	15%	−10%	25%	135%	−100%	2%
Random template	5	210	78	29	65	58	0	68

Example of random percentages generated by the built-in self-random test (Section 2.5).

**Table 7:** INNSZ experimental data.

No.	Concept	Jan-08	Apr-08	Aug-08	Dec-08	Jan-09	Apr-09	Aug-09	Dec-09	Jan-10	Apr-10	Aug-10	Dec-10
1	Patients	**6**	**7**	**7**	**4**	**9**	**118**	**87**	**10**	**18**	**20**	**10**	**10**
2	Available beds	38	38	38	38	39	39	39	39	40	40	40	40
3	Available areas	68	68	68	68	68	68	68	68	68	68	68	68
4	Hemodynamic monitors	33	33	33	33	33	33	33	33	70	70	70	70
5	Mechanical ventilators	52	52	52	52	76	76	76	76	76	76	76	76
6	Doctors	25	25	25	25	27	27	27	27	29	29	29	29
7	Respiratory technicians	13	13	13	13	16	16	16	16	16	16	16	16
8	Nurses	67	70	75	71	71	72	75	82	76	72	77	73
9	OSRDI-A	80	80	80	80	62	62	62	62	46	46	46	46
10	OSRDI-B	47	55	55	31	69	907	669	76	135	150	75	75
11	OSRDI	**80**	**80**	**80**	**80**	**69**	**907**	**669**	**76**	**135**	**150**	**75**	**75**

Data used by OSRDI model (2008–2010). Concept: variable used to determine saturation index. OSRDI-A and B. Source: National Institute of Medical Sciences and Nutrition Salvador Zubiran (INNSZ) in Mexico City.

**Table 8:** OSRDI model warnings.

Quotients	Built-in self-random test	OSRDI
Beds/medical personal	13	13
Available areas/equipment	8	7
Beds/equipment	5	5
Patients/beds	5	5

Built-in self-random test and OSRDI model warning matches.
